# Why do some asthma patients respond poorly to glucocorticoid therapy?

**DOI:** 10.1016/j.phrs.2020.105189

**Published:** 2020-10

**Authors:** Ishbel Henderson, Elisabetta Caiazzo, Charles McSharry, Tomasz J. Guzik, Pasquale Maffia

**Affiliations:** a Institute of Infection, Immunity and Inflammation, College of Medical, Veterinary and Life Sciences, University of Glasgow, Glasgow, United Kingdom; b Department of Pharmacy, University of Naples Federico II, Naples, Italy; c Institute of Cardiovascular and Medical Sciences, College of Medical, Veterinary and Life Sciences, University of Glasgow, Glasgow, United Kingdom; d Department of Internal Medicine, Jagiellonian University, Collegium Medicum, Kraków, Poland

**Keywords:** AP1, activator protein-1, CSF 3, colony-stimulating factor 3, FE_NO_, fractional exhaled nitric oxide, FEV_1_, forced expiratory volume in 1 second, GCs, glucocorticosteroids, GR, GC receptor, GRE, GC response elements, GWAS, genomic wide association studies, HDAC, histone deacetylase, HPA, hypothalamic-pituitary-adrenal, IAV, influenza A virus, ICS, inhaled corticosteroids, IL, interleukin, JNK, c-Jun N-terminal kinase, MAP, mitogen-activated protein, MAPK, mitogen-activated protein kinase, NF-κB, Nuclear factor-κB, NLRP3, NLR Family Pyrin Domain Containing 3, PBMCs, peripheral blood mononuclear cells, RSV, respiratory syncytial virus, TF, transcription factors, Th, T helper, VDBP, vitamin D-binding protein, Asthma, Glucocorticoids, Glucocorticoid receptor, Steroid-resistant asthma

## Abstract

Glucocorticosteroids are the first-line therapy for controlling airway inflammation in asthma. They bind intracellular glucocorticoid receptors to trigger increased expression of anti-inflammatory genes and suppression of pro-inflammatory gene activation in asthmatic airways.

In the majority of asthma patients, inhaled glucocorticoids are clinically efficacious, improving lung function and preventing exacerbations. However, 5–10 % of the asthmatic population respond poorly to high dose inhaled and then systemic glucocorticoids. These patients form a category of severe asthma associated with poor quality of life, increased morbidity and mortality, and constitutes a major societal and health care burden. Inadequate therapeutic responses to glucocorticoid treatment is also reported in other inﬂammatory conditions such as rheumatoid arthritis and inflammatory bowel disease; however, asthma represents the most studied steroid-refractory disease. Several cellular and molecular events underlying glucocorticoid resistance in asthma have been identified involving abnormalities of glucocorticoid receptor signaling pathways. These events have been strongly related to immunological dysregulation, genetic, and environmental factors such as cigarette smoking or respiratory infections. A better understanding of the multiple mechanisms associated with glucocorticoid insensitivity in asthma phenotypes could improve quality of life for people with asthma but would also provide transferrable knowledge for other inflammatory diseases. In this review, we provide an update on the molecular mechanisms behind steroid-refractory asthma. Additionally, we discuss some therapeutic options for treating those asthmatic patients who respond poorly to glucocorticoid therapy.

## Graphical abstract

**Figure:**
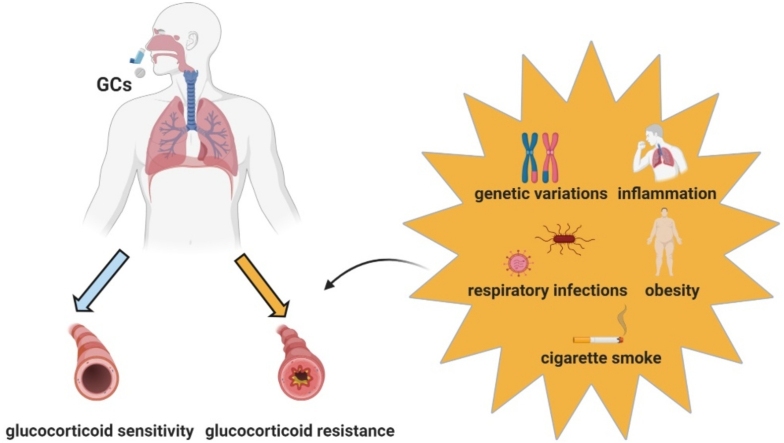

## 1 Introduction

Glucocorticosteroids (GCs), also called glucocorticoids, corticosteroids or steroids, are natural regulators of a wide range of biological processes including the hypothalamic-pituitary-adrenal (HPA) axis, immunity and energy metabolism, primarily to maintain homeostasis. In humans, the hormone cortisol is the primary endogenous glucocorticoid, synthesized and secreted by the adrenal cortex. It interacts with the GC receptor (GR) to regulate a plethora of signaling pathways [1]. GCs are associated with potent anti-inflammatory activity, which can be exploited for therapeutic drug use. Synthetic GCs (e.g. prednisolone, dexamethasone) can be synthesized in bulk and designed for higher affinity binding to GR. They are used in medicine, to mimic this natural pathway of immune suppression and attenuate inappropriate inflammation. They are the mainstream therapy for a wide range of acute and chronic inflammatory diseases, including asthma. The Global Initiative for Asthma (GINA) defines asthma as “a heterogeneous disease that is characterized by chronic inflammation of the airways and a clinical history of wheezing, cough, tightness of chest and shortness of breath varying with time and intensity, as well as expiratory airflow limitation” (https://ginasthma.org/). Asthma affects over 300 million people worldwide, with increasing incidence. The heterogeneity of ‘asthma’ suggests it should be an umbrella term for different phenotypes. There are two recognizable endotypes: allergic asthma, driven by a T helper 2-subset (T2-type) response, or non-allergic, non-atopic (non-T2) asthma, driven by other immune cells such as neutrophils [[2], [3], [4]]. GCs represent first-line treatment for asthma but there are two major limitations: firstly, toxic high dose-dependent side effects and secondly the refractory response seen among asthma patients, predominantly those with severe disease. Early asthma studies, mostly in children, characterized asthma as an IgE-dependent inflammatory response to allergens associated with eosinophilia [5]. This led to treatments centralized around inhibiting Th2 cytokines, and this delayed the recognition and understanding of non-T2 asthma phenotypes and hindered development of appropriate treatment options. T2 asthma is the dominant sub-group, and generally responds effectively to GC treatments. In contrast, non-T2 severe asthma is associated with GC insensitivity.

Herein we provide a state‐of‐the‐art overview of the proposed mechanisms leading to glucocorticoid refractory asthma and potential therapeutic strategies.

## 2 Mechanisms of action of glucocorticoids

**Fig. 1:**
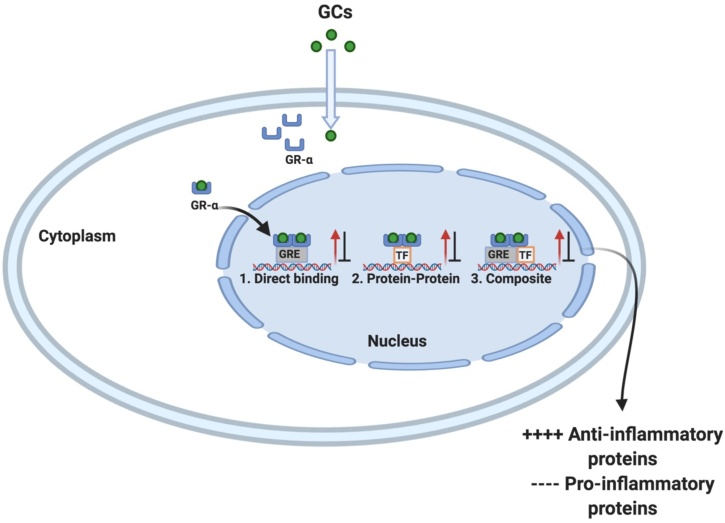
Glucocorticoid transcriptional regulation. The lipid-soluble glucocorticoid (GC) passively diffuses through the cell membrane and binds to the glucocorticoid receptor (GR-α) in the cytoplasm. Once ligated the GC/GR complex translocate into the nucleus through the nuclear pore or exerts non-genomic effects in the cytoplasm. In the nucleus, the complex can either: 1. Bind directly to DNA and regulate transcription using positive GC response elements (GREs) or negative GREs; 2. Interact with other transcription factors (TF) to mediate their activity or; 3. Compositely bind to both DNA and TFs to regulate gene transcription. Notably, all three of these mechanisms can positively and negatively regulate gene expression. Figure created in BioRender (https://biorender.com/).

Although GCs have been widely used for many decades, the complete understanding of their multiple molecular mechanisms of immune modulation is still elusive. It is known that they act through genomic and nongenomic mechanisms. The strong suppression of airway inflammation is mainly due to the genomic mechanism. GCs act by binding to intracellular receptors of the target cell (glucocorticoid receptors; GRs). Genomic mechanisms derive from glucocorticoids binding to glucocorticoid receptors in the cytoplasm and the translocation of the GC/GR complex into the nucleus. In the nucleus, the GC/GR complex modifies transcription of specific genes through direct DNA binding or transcription factor inactivation (Fig. 1). This is either done by binding to small motifs known as GC response elements (GRE) in the promoter regions of susceptible genes. GRs consist of different subunits, with a variable N-terminal domain, C-terminal domain, and a DNA binding domain, with zinc fingers to assist genomic interactions. GRs are located in an inactive form in the cytoplasm, as a multi-protein complex, attached to a chaperone protein. Originally, upon activation, it was believed that the GR and chaperone protein dissociated allowing the GR to translocate into the nucleus. However, research now indicates that the chaperone complex is required for nuclear transportation [6]. The GR can function as a monomer, homo, or hetero-dimer, and recently described as a tetramer [7]. There are two variants of the receptor, GR-α, and GR-β, with subtle splicing differences in the C-terminal domain. The GR-β isoform is most abundantly expressed; however, it is unable to bind to GCs and therefore cannot transduce GC-induced functions. It is believed to regulate GC activity, through antagonizing the GR-α isoform and regulation through GR-α/β heterodimers [8,9]. GR-α is subject to different post-translational modifications, which include phosphorylation, acetylation and other modifications that affect GR signaling pathways [10].

Once inside the nucleus, the GC/GR complex functions by regulating up to 20 % of genes expressed by immune cells. Specifically, GCs act by trans-repressing inflammatory genes and stimulating the transcription of anti-inflammatory genes leading to reduced activation, recruitment and survival of inflammatory and epithelial cells [1,11,12]. Glucocorticoids may also regulate the immunomodulatory function of smooth muscle cells and affect airway remodeling in asthma [13]. Other genomic mechanisms such as regulation of mRNA stability have been also described [14]. Nongenomic actions are mediated by specific interaction with membrane-bound or cytoplasmic GRs, or nonspecific interactions with the cell membrane [15].

Synthetic GCs are designed with optimal characteristics for potent, high affinity binding to the GR only, making them more specific than natural GC, which bind to both the GR and the closely related mineralocorticoid receptor [16,17]. GCs also possess pro-inflammatory effects, under stress conditions [18].

## 3 Glucocorticoid resistance

The term glucocorticoid resistance is formally used to describe the resistance to adrenal suppression by dexamethasone, as in Cushing’s syndrome. In asthma, patients generally respond well to GCs, but in some this can vary and when responses to inhaled and then oral GCs are inadequate this is called steroid unresponsive, refractory or resistant asthma. According to international guidelines, for adults and children patients with persistent asthma low-dose inhaled corticosteroids (ICS) with or without long-acting bronchodilator (β_2_-agonists) represent the ﬁrst-line therapy. Besides, they are recommended in patients with intermittent or mild asthma requiring a short-acting β_2_-agonist more than twice a week or twice a month (https://ginasthma.org/).

For the majority of asthma patients, ICS work well, improving lung function and reducing exacerbations. Patients with difficult asthma require higher doses of oral GCs to manage their asthma, and these include 5–10 % of patients who don’t respond satisfactorily to these drugs, resulting in difficult to manage asthmatic symptoms thus denoted as having steroid-resistant asthma [19]. Th2-low asthma phenotypes are less responsive to steroid therapies and have a higher prevalence of severe asthma cases. However, glucocorticoid insensitivity in patients with persistent eosinophil inflammation has also been described [20]. They are a disproportionately large burden on health care; the 10 % with refractory asthma cost 80 % of health care costs [21], along with a significantly increased morbidity and mortality [22]. This emphasize the importance of a better understanding of this poor responsiveness and the need for new therapeutic options.

Steroid resistance is defined as <15 % improvements in forced expiratory volume in 1 s (FEV_1_), after 2 weeks of appropriate dose steroid treatment. These patients, however, do respond well to β_2_-adrenergic agonist-mediated vasodilation [23,24]. There are currently no clinically accepted biomarkers or phenotypes for resistance; meaning diagnosis is based on the clinical history and lung function after sufficient steroid treatments. This results in patients receiving increasing doses of steroids for extended periods, until it is recognized that this is ineffective for treating the severity of asthma. Indeed, a key cause of morbidity in these patients comes from the toxic side effects of long-term high-dose steroids; these include increased susceptibility to infections, osteoporosis, hyperglycemia and cardiovascular disease [25,26].

### 3.1 Understanding mechanisms of the inadequate responses to glucocorticosteroids

To gain a better understanding of steroid resistance an important distinction must be made between patients who poorly manage their asthma and the group of genuinely refractory asthmatic patients. Several studies highlighted that about one third of difficult asthma cases that did not respond to CS was due to poor compliance and not steroid insensitivity [27,28]. The clinical pattern of exacerbations and poor asthma control was similar to the GC-refractory patients. Fortunately, poor-compliance can be assessed by measuring fractional exhaled nitric oxide (FE_NO_) levels which is high in asthma and is reduced confirming compliance with steroid treatment unless the patient is bluntly resistant to the treatment [29].

Successful adherence to medications is a complex issue, with many patients either self-assessing their condition and coming off long-term treatments when they feel healthy or become overly concerned about the side effects of the treatments. Another major issue is poor inhalation technique, considered non-intentional non-adherence [30], which can be resolved with better training. Once the true refractory patient cohort is identified studies can be more accurate, focusing on the causes of insensitivity and alternative therapeutic interventions.

Refractory patients have typically severe asthma, with low percent-predicted FEV_1_ indicating higher levels of fixed airway obstruction and reduced lung function [31,32]. A consideration here is whether the lack of efficacy of GCs results in severe symptoms or whether the inflammatory mechanisms maintaining severity also drives resistance.

### 3.2 Cellular and molecular basis of glucocorticoid-resistance

Poor steroid responsiveness can be inherited or acquired. Inherited genetic mutations specifically associated with refractory asthma remain poorly described [33]. Many genomic wide association studies (GWAS) and pharmacogenomic studies have been carried out to investigate the relationship between genetic variations and response to steroids. High-density oligonucleotide microarray studies of peripheral blood mononuclear cells (PBMCs) from patients with glucocorticoid-sensitive asthma and those with glucocorticoid-resistant asthma revealed that 11 genes accurately predicted corticosteroid resistant asthma [34]. Further pharmacogenomic studies including an appropriately large patient cohort would be useful to power a study to fully differentiate glucocorticoid-resistant and glucocorticoid-sensitive asthmatic patients. Single nucleotide polymorphisms in the GLCCI1 gene, encoding the glucocorticoid-induced transcript 1 protein, were associated with response to GC therapy in asthma [35]. These findings were replicated in multiple candidate gene analysis studies [36,37]. The functional single nucleotide polymorphism, rs37973, was associated with reduced ICS sensitivity. Children with two copies of the mutant allele are less responsive to GC therapy [35], and increased expression of GLCCI1 demonstrated better responses to ICS [38]. Several additional genes have significant associations with GC insensitivity. However, these finding were not well replicated between studies, perhaps due to variation in protocols, heterogeneity of asthma phenotype, or the complexity of the GC pathways. It is feasible that GC insensitivity is not caused by a singular mutation, and more likely involves a range of genetic variations that remain to be determined.

Multiple molecular mechanisms have been identified associated with GC dysfunction including: reduced GR-α expression [39], defective binding between the GC and the GR or between the GR complex and DNA [40], and increased antagonism, either from increased pro-inflammatory transcription factors or increased GR-β expression [41]. Additionally, GR phosphorylation by e.g. p38 mitogen-activated protein kinase (MAPK) and by reduced activity of histone deacetylase 2 (HDAC2) can reduce the expression various anti-inflammatory genes induced by GCs [42,43].

As examples of acquired GC resistance, inflammation or oxidative stress can negatively affect GR signaling [22] Possible mechanisms for acquired resistance could be related to immune dysregulation; for example, interleukin (IL)-2, IL-4 and IL-13 are often overexpressed in the lungs of steroid insensitive patients [[44], [45], [46]]. This profile of cytokine up-regulation is associated with reduced GR affinity *in vitro* through activation of p38 mitogen-activated protein kinase resulting in the phosphorylation of GR and diminished nuclear translocation in inflammatory cells [47].

Th1 cytokines have also been associated with GC-resistance. Specifically, IFN-γ can increase GR phosphorylation and inhibit GR nuclear translocation in different experimental models of steroid-resistant airway hyperresponsiveness by up-regulating miR-9 expression in the lung and pulmonary macrophages [48]. Recently, TNF-α and IFN-γ cytokines have been shown to sustain glucocorticoid-resistance in human fetal airway smooth muscle cells by promoting the Nuclear factor-κB (NF-κB) pathway and Stat1 phosphorylation [49].

Kim and co-workers have suggested that in murine models of steroid-resistant allergic airway disease, the exaggerated NLR Family Pyrin Domain Containing 3 (NLRP3) inflammasome/IL-1β activation critically contributed to glucocorticoid resistance [50]. The mechanism is not yet fully understood but included a role for IL-1β in Th17 cell differentiation and IL-17 production [51]. It is interesting to note that asthmatic patients resistant to glucocorticoids show increased Th17 cells and IL-17A levels [52], and the adoptive transfer of Th17 cells in mice has resulted in the development of steroid insensitivity [53]. Accordingly, Th17 responses have been shown to increase the expression of GR-β, a mechanism of steroid resistance in bronchial epithelial cells [54]. Very recently, Ouyang et al. have found that IL-17A synergizes with dexamethasone in inducing colony-stimulating factor 3 (CSF 3) in both human airway smooth muscle cells and fibroblasts through transcriptional and post-transcriptional regulation. This effect is further increased in the presence of TNF-α, as described above, and has been associated with glucocorticoid resistance [55]. Additionally, it has been suggested that IL-17 produced in the lung by type 3 innate lymphoid cells, ILC3, may play a role in steroid resistance associated with the obesity phenotype of asthma [56].

GC insensitivity has been associated with dysregulated IL-10 production. Specifically, T lymphocytes from corticosteroid-resistant asthmatic patients have impaired IL-10 production following *in vitro* stimulation with dexamethasone compared to T lymphocytes from steroid-sensitive asthmatics [57]. This defect can be reversed by the combination of GC with salmeterol or the administration of vitamin D3 [58,59]. Recently, a decreased regulatory T-cell activity has been shown in older asthmatic patients that render them more vulnerable to type 2 inflammation and steroid resistance [60]; thereby, suggesting age as an important factor in determining glucocorticoid sensitivity.

Exogenous factors such as cigarette smoking, respiratory viral and bacterial infections, high-fat diet and/or obesity, may also contribute to mechanisms of steroid-resistant asthma. The airway inflammation observed in asthma patients who smoke cigarettes is typically neutrophilic rather than eosinophilic. This non-T2 endotype is consistent with the major phenotypes of steroid-refractory asthma. A mechanism proposed for cigarette smoke induced GC-insensitivity is a reduced ratio of GR-α to GR-β isoforms [61], resulting in increased antagonism of the GR-α. Cigarette smoke is associated with reduced HDAC activity in alveolar macrophages, which controls access to chromatin, a crucial stage in GC-mediated gene regulation [62,63]. This could be a potential target for novel therapies, to re-balance the isotype ratio and increase HDAC activity to re-sensitize patients to steroids. An additional mechanism included the ligation by cigarette smoke components of the aryl hydrocarbon receptor that suppresses smoke-induced inflammation, apoptosis and oxidative stress. It is proposed this is done through microRNA regulation inhibiting protein synthesis [64] and indirectly regulating the Th17 pathways, which have been linked with steroid resistance, as discussed above. Th17 cells mediate neutrophilic airway inflammation by stimulating the production of IL-8. McSharry et al. have shown the increase in neutrophils and IL-8 levels in sputum fluid from asthmatic smokers compared to that from nonsmokers with asthma, suggesting a contribution for this cytokine in glucocorticoid insensitivity [65]. Another clinically important study has shown that steroid sensitivity returns after smoking cessation suggesting mechanisms of resistance are reversible [66] and providing smoking cessation advice as a clear therapeutic strategy.

Exposure to combustion products from cigarette smoke or burning biomass fuel is a major cause of the development of chronic obstructive pulmonary disease (COPD). COPD is stubbornly refractory to mainstay corticosteroid treatment and the molecular mechanisms of steroid insensitivity in COPD are incompletely understood [67]. Similar to asthma in cigarette smokers, it has been proposed that cigarette smoke and oxidative stress in COPD may decrease HDAC2 activity [22] and increase various kinase pathways such as p38 MAPK [68]. Of note, the lung inflammation in COPD and smokers with asthma is predominantly neutrophilic [69], and is the basis of defining an asthma−COPD overlap syndrome (ACOS) sharing steroid-refractory Th17 endotype that predisposes to neutrophilia and neutrophilic asthma is associated to steroid resistance [70,71].

A role for viral and/or bacterial respiratory infections in glucocorticoid refractivity in asthmatic patients has been also described. Specifically, *Chlamydia pneumoniae*, *Haemophilus influenzae*, rhinovirus, influenza A virus (IAV) and respiratory syncytial virus (RSV) infections have each been associated with steroid resistance [[72], [73], [74], [75], [76]]. The reduction of GR-α nuclear translocation through NF-κB and c-Jun N-terminal kinase activation has been proposed as a molecular mechanism of glucocorticoid insensitivity in rhinovirus-infected primary human bronchial epithelial cells [77].

Non-T, neutrophilic asthma patients often have bacterial infections, which could impair steroid sensitivity [78]. Some bacterial products, such as staphylococcal endotoxins B, have been shown to increase GR-β expression [79]. Recently, Kim et al. have developed novel mouse models of steroid-resistant asthma driven by bacterial (Chlamydia and Haemophilus influenzae) and viral (influenza and RSV) respiratory tract infections. In these experimental models, the authors demonstrated a role for miR-21 in inducing steroid insensitivity through PI3K-mediated phosphorylation and nuclear translocation of pAKT [80].

Steroid resistance has been also associated with fungus-exposed patients through induction of Th2/Th17 responses [81]. Also Aspergillus alternata exposure has been shown to induce IL-33 dependent steroid-resistant asthma, mediated by ILC2 and Th2 cells in neonatal mice [82]. It has been suggested that the ability of IL-33 to activate p38-MAPK in CD4 + T cells and to induce phosphorylation of GR may be a mechanism underlying glucocorticoid insensitivity [83].

As described above, GCs can exert also non-genomic actions, especially at high concentrations, and few non-genomic pathways have been identified to date. GCs can inhibit the degranulation of mast cells through stabilization of the plasma membrane or by a reduction in [Ca2+] elevation [84]. In addition, GCs can exert their anti-inflammatory effects by negative interference with MAPK signaling pathways [85]. Abnormalities of these non-genomic mechanisms on immune cells may contribute to GC insensitivity; however, more research is needed to fully understand how non-genomic mechanisms can influence GC sensitivity.

**Fig. 2:**
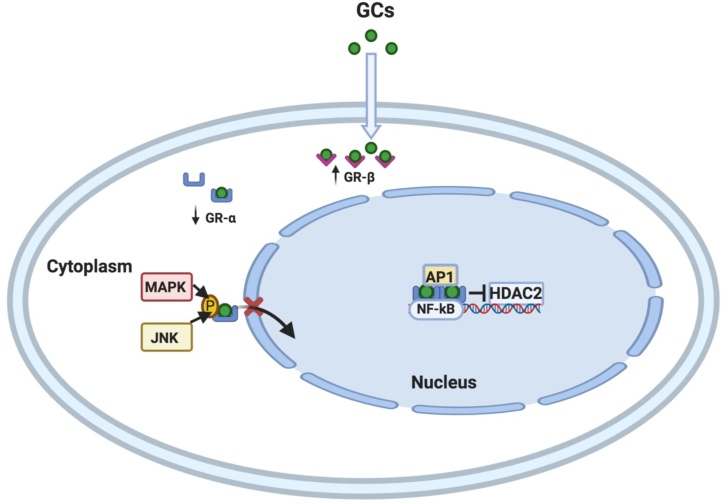
Cellular mechanisms behind glucocorticoid resistance. Glucocorticoid resistance can be associated to reduced expression of GR-α that mediate the pharmacological actions of glucocorticoids, increased expression of negative isoform GR-β; reduced GR-α translocation into nucleus due to hyperphosphorylation of GR-α p38 by kinases such as p38 MAPK and JNK; increased expression of inflammatory transcription factors like NF-kB or AP-1 that compete for DNA binding; inhibition of HDAC2 activity that suppresses various inflammatory gene expression. Figure created in BioRender (https://biorender.com/).

Key molecular mechanisms involved in steroid resistance in asthmatic patients are summarized in Table 1 and Fig. 2.

**Table 1:** Proposed molecular mechanisms of steroid resistance.

Mechanism	References
Genetic abnormalities in GRs	[83]
Reduced GR-α expression	[39]
Defective GC binding to GR-α	[40]
Reduced GR-α translocation due to increase phosphorylation by kinases such as p38 MAPK and JNK	[43]
Reduced HDAC2 activity and expression	[42]
Increased pro-inflammatory transcription factor activation, such as NF-kB and AP1	[41]
Increased GR-β expression	[86]

Abbreviations: GR: glucocorticoid receptor, GC: glucocorticoid, MAPK: mitogen-activated protein kinase, JNK: c-Jun N-terminal kinase, HDAC: histone deacetylase, NF-KB: nuclear factor-κB, AP1: activator protein-1.

### 3.3 Therapeutic perspectives

From the information provided, and the little progress in the past decades towards effective treatments for non-T2, steroid-refractory asthma, it is clear that future research needs a multidisciplinary approach and knowledge collaborations to reach a deeper understanding of steroid insensitivity. Some of the main molecular mechanisms have been identified; however, a deeper understanding of these mechanisms is needed before pharmacogenomic data can be clinically utilized in predicting drug responses and effectively optimizing treatments.

Innovative solutions should consider environmental and genetic factors contributing to the resistance, better technologies for molecular imaging of inflammation [87], and, in the era of big data, the establishment of large databases to support immune research into drug discovery and therapeutics [88,89], along with omics approaches for the identification of biomarkers. Several serum biomarkers have been proposed to predict steroid resistance in asthmatic children such as vitamin d-binding protein (VDBP), miRNA-21 and OX40 ligand [[90], [91], [92], [93]].

The development of more personalized approaches for steroid-resistant asthma is particularly useful because of the different mechanisms and multiple immunological and inflammatory phenotypes likely leading to steroid resistance. Traditionally steroids were considered a universal treatment, with reduced sensitivity forcing a higher dose regime, following the theory that stronger Th2 responses require higher doses of steroid suppression. It is now evident that complex asthma phenotypes highlight the need for patient stratification and individualized therapy.

Identifying the underlying endotype driving each phenotype should be utilized when considering the optimal treatments. This requires multidisciplinary collaborations to identify the different aspects of insensitivity. Once a better understanding is gained optimal treatments, such as biologicals, can be used instead of non-specific immunosuppressants.

Better education should also be provided on steroid functions and inhaler technique, to limit the non-adherence patients. Most steroid-resistant research is done on asthma; however, similar insensitivity is observed, with more frequent rates, in other inflammatory respiratory diseases such as chronic obstructive pulmonary disease, and in rheumatoid arthritis, and inflammatory bowel disease [94,95]. A better understanding of multiple mechanisms associated with GC insensitivity in asthma could provide transferrable knowledge for other inflammatory diseases since similar molecular mechanisms have been proposed. To attain more accurate research a new disease model is required, to fully understand the mechanisms, as well as identifying biomarkers to use for early identification and screening for insensitivity. This will help alleviate the time lost trialing different steroid doses, as well as optimally treating the patient.

Most clinical trials to validate new therapeutics for asthma typically exclude smokers. Since steroid insensitivity is prominent in smokers [96], future clinical trial studies should include this category of asthma patients.

Therapeutic target for reversing steroid insensitivity could include blocking the underlying mechanisms e.g. with antibodies against the key cytokines such as IL-17, IL-8, and TNF-α associated with the neutrophilic airway inﬂammation that is strongly steroid-resistant. Regarding NLRP3 inflammasome/IL-1β, it has been demonstrated that specific inhibition of the NLRP3 inflammasome is more advantageous than global inhibition of IL-1β [79].

Interestingly, the combined use of different drugs can restore glucocorticoid sensitivity. Steroid-resistance in asthma has been associated with imbalanced acetylation and deacetylation of GRs variously regulating gene transcription. Increased HDAC activity using theophylline, PI3K and p38 MAPK inhibitors [[96], [97], [98]] may be beneficial, especially in glucocorticoid resistant asthmatic smokers. Combined therapy with long-acting beta 2 agonists has improved glucocorticoid responses by affecting GR translocation and phosphorylation. This therapeutic strategy and could be useful for asthmatic patients in whom a poor response is related to abnormal GR signaling [99,100]. Other studies suggest that macrolides such as azithromycin and clarithromycin potentiate glucocorticoid sensitivity in asthma, but the mechanism remains unclear [[101], [102], [103]]. Another interesting study has shown that statins increase the anti-inflammatory effect of glucocorticoid though induction of indoleamine 2, 3-dioxygenase in alveolar macrophages [104]. A combination of these different therapeutic strategies may help to effectively reduce GC resistance.

Finally, recent new highly potent glucocorticoids have been developed for steroid-resistant severe asthma. Among them, only GCVSG158 was demonstrated to reverse steroid-resistance in a murine model of eosinophilic and neutrophilic airway inflammation [105].

## 4 Conclusions

In conclusion, although glucocorticoid resistance is observed in a small proportion of asthmatic patients, it represents a serious clinical and socioeconomic problem. Therefore, future research on the molecular mechanisms of multiple steroid-resistant asthma endotypes and the identification of subgroups of patients with poor responses to steroids will facilitate the selection of appropriate treatment, in a stratified fashion for those phenotypes and the development of novel therapeutic approaches.

## Declaration of Competing Interest

The authors declare that they have no conflicts of interest.
